# Fe/Ni Bimetallic Organic Framework Deposited on TiO_2_ Nanotube Array for Enhancing Higher and Stable Photoelectrochemical Activity of Oxygen Evaluation Reaction

**DOI:** 10.3390/nano10091688

**Published:** 2020-08-27

**Authors:** Sheng-Mu You, Waleed M. A. El Rouby, Annadurai Thamilselvan, Cheng-Kuo Tsai, Win Darmanto, Ruey-An Doong, Pierre Millet

**Affiliations:** 1 Institute of Environmental Engineering, National Chiao Tung University, 1001 University Road, Hsinchu 30010, Taiwan; sheng-mu.you@universite-paris-saclay.fr or; 2 ICMMO-Eriée, UMR CNRS 8182, Paris-Saclay University, 91400 Orsay, France; 3 Materials Science and Nanotechnology Department, Faculty of Postgraduate Studies for Advanced Science (PSAS), Beni-Suef University, 62511 Beni-Suef, Egypt; waleedmohamedali@psas.bsu.edu.eg; 4 Institute of Analytical and Environmental Sciences, National Tsing Hua University, 101, Sec. 2, Kuang Fu Road, Hsinchu 30013, Taiwan; tamilselvan8033@gmail.com; 5 Department of Safety Health and Environment, National Yunlin University of Science and Technology, Yunlin 64002, Taiwan; cktsai@yuntech.edu.tw; 6 Department of Biology, Faculty of Science and Technology, Airlangga University, Surabaya 60115, Indonesia; windarmanto@fst.unair.ac.id; 7 Faculty of Science and Technology, Airlangga University, Surabaya 60115, Indonesia

**Keywords:** titanium dioxide nanotube array (TNTA), bimetallic organic framework, water oxidation, oxygen evolution reaction (OER), photoelectrocatalyst

## Abstract

Photoelectrochemical (PEC) water splitting is a promising strategy to improve the efficiency of oxygen evolution reactions (OERs). However, the efficient adsorption of visible light as well as long-term stability of light-harvesting electrocatalysis is the crucial issue in PEC cells. Metal–organic framework (MOF)-derived bimetallic electrocatalysis with its superior performance has wide application prospects in OER and PEC applications. Herein, we have fabricated a nickel and iron bimetallic organic framework (FeNi-MOF) deposited on top of anodized TiO_2_ nanotube arrays (TNTA) for PEC and OER applications. The FeNi-MOF/TNTA was incorporated through the electrochemical deposition of Ni^2+^ and Fe^3+^ onto the surface of TNTA and then connected with organic ligands by the hydrothermal transformation. Therefore, FeNi-MOF/TNTA demonstrates abundant photoelectrocatalytic active sites that can enhance the photocurrent up to 1.91 mA/cm^2^ under 100 mW/cm^2^ and a negligible loss in activity after 180 min of photoreaction. The FeNi-MOF-doped photoanode shows predominant photoelectrochemical performance due to the boosted excellent light-harvesting ability, rapid photoresponse, and stimulated interfacial energy of charge separation under the UV-visible light irradiation conditions. The results of this study give deep insight into MOF-derived bimetallic nanomaterial synthesis for photoelectrochemical OER and provide guidance on future electrocatalysis design.

## 1. Introduction

The oxygen evolution reaction (OER) is the half-reaction of the water oxidation process, which has attracted great research attention in recent years due to its significant role in various energy conversion and storage technologies [1,2]. Since the kinetics of the OER are drowsy, which is caused by intricate proton-couple electron transferring, precious and rare metal-containing catalysts such as Pt, Au, Ru, and Ir are usually required to facilitate high activity and promote reaction efficiency [3,4]. Due to the limited amount and high cost issues of precious metals, the abundant materials in the earth such as iron, nickel, cobalt, and titanium composites are considered as part of the current goal to develop an efficient system without consuming precious metals [5,6,7,8]. Therefore, the search for active, stable, and cost-efficient non-precious metal-based electrocatalysts for hydrogen evolution via water splitting is urgently needed to make a substantial improvement on energy technologies. In particular, photoelectrochemical (PEC) devices have recently been regarded as a promising method of converting solar to hydrogen energy and the PEC water oxidation using light- harvesting semiconductor- like TiO_2_ nanomaterials [9,10,11] and metal– organic frameworks (MOFs) [12,13,14,15].

MOFs are kinds of porous and crystalline compounds that consist of metal ions or clusters coordinated to organic ligands to form one-, two-, or three-dimensional structures [14,16,17,18,19]. They are a subclass of polymers with the special feature of microporous architectures, and they are regarded as a potential material for gas storage, purification, and separation, as well catalysis and energy applications [19,20,21]. Conjugated carboxylates-based MOFs, such as ZIF-67 [22], MOF-74 [23], HKUST-1 [24], MIL-101 [25], and MOF-5 [26], are well-known materials that are employed in energy application for their large gas adsorption capacity, and they have attracted a tremendous attention because of their ultra-large surface area, tunable pore texture, and specific electrochemical catalytic performance [27]. Recently, the fabrication of multi-dimensional MOFs nanocomposites has been done via various synthesis procedures for the application of water splitting. As a result of the tunable architecture of MOF nanocomposites, some researches have demonstrated the superior performance on the water-splitting chemistry including hydrogen production, oxygen evolution reaction (OER) and overall oxidation process [13,14]. However, the rapid recombination of electrons generated from MOFs and the fast oxidization by charge transfer are the main problems for PEC systems. Hence, a recent study has demonstrated that a great enhancement on the performance of a water-splitting PEC could be achieved by using a surface-modified ZIF-67 MOF-derived semiconductor, which contains highly porous cobalt oxide (CoO_x_) and BiVO_4_ co-catalysts, as the photoanode to accelerate the kinetics of water oxidation rather than serving as a surface passivation layer [28].

Titanium-based materials are considered as good photoelectrocatalysts because of their highly efficient photoconversion capability, longevity, and cost–benefit based on earth-abundant metals [29]. In the past decades, several studies have focused on their various dimensional morphologies to enhance the efficiency of their specific applications [9,10,30]. Those morphologies of TiO_2_ include nanoparticle, nanotube, nanotube array, nanoflower, nanofiber, nanorods, and their nanocomposites [31,32,33,34,35], which increase the performance of different fields. Based on the electron transfer theory, a nanoarray structure can promote electron flow smoothly, providing a large active surface area to increase the activity and accelerate the surface reactions. Moreover, the open space between nanoarrays can facilitate the diffusion of active species readily, and the direct contact of nanoarrays to the underneath conductive substrate guarantees the electron transportation as well as immobilizes the active material on the electrode surface [36]. Moreover, the surface area and porosity of the photosensitizer are significant as well for the performance of catalysis in PEC cell [37]. Hence, TiO_2_ nanotube array structures for photoelectrochemical applications can be easily found in several studies [32,38]. However, TiO_2_-based photoanodes suffer from two major limitations: (1) no absorption of light in the visible region due to a relatively large band gap (3.2 eV for anatase; 3.0 eV for rutile), and (2) the fast recombination of excitons due to the short diffusion paths of charge carriers [33,39]. As the result of those defects of materials, the modification of TiO2-based photoanodes with other dopants or nanomaterials is necessary to apply for photoelectrochemical reactions [31,33]. Thus, bimetallic MOFs such as Co/Ni [40], Fe/Zn [41], and Fe/Co [42] have been synthesized to promote the production of the photocurrent as well as to enhance the electrons transfer and conductivity [43,44,45]. Especially, the nickel and iron bimetallic organic framework (FeNi-MOF) or FeNi-layered double hydroxide (LDH) has been reported to greatly boost the PEC water oxidation, resulting from the synergistic effect of Ni and Fe active sites. They have been considered as ideal materials for PEC water oxidation [46,47,48]. However, the application of a bimetallic MOF with a TiO_2_ nanotube array (TNTA) for OER has received less attention.

Herein, we have reported an efficient Fe/Ni bimetallic organic framework (FeNi-MOF) to boost the photoelectrocatalytic activity of FeNi-MOF/TNTA for OER application. The Fe/Ni-incorporated bimetallic MOF was synthesized by the electrodeposition method via cyclic voltammetry and then deposited on TNTA grown on Ti substrate (FeNi-MOF/TNTA). Subsequently, the anchored iron and nickel onto TNTA produced the metal hydroxide layer, and then the organic ligand, trimethyl 1,3,5-benzenetricarboxylate (BTC), was added with dimethylformamide (DMF) and methanol in specific ratio to transform Fe and Ni hydroxide into MOF crystalline particles. In addition, the bimetallic MOF surface morphology and Fe/Ni oxidation behavior were analyzed by scanning electron microscope (SEM), transition electron microscope (TEM), and X-ray photoelectron spectroscopy (XPS). Moreover, the incorporation of Fe/Ni with TNTA significantly promotes the charge transfer kinetics and makes the close contact between the surface-active sites and reaction medium, enabling the excellent electrocatalytic performance of FeNi-MOF/TNTA on photoelectrochemical oxygen evaluation application. Additionally, FeNi-MOF/TNTA significantly enhances the photocurrent response to 1.40 mA cm^−2^ under 100 mW cm^−2^ UV-visible light irradiation.

## 2. Materials and Methods

### 2.1. Chemicals

All the chemicals were of analytical grade, and distilled water (18.2 Ω cm) was used throughout all the experiments. A titanium plate (99.9%) was purchased from M&T Co., Ltd. (Hsinchu, Taiwan), iron(III) nitrate hydrate (99%), nickel(II) nitrate hexahydrate (98%), trimethyl 1,3,5-benzenetricarboxylate (98%), ammonium fluoride (98%), and ethylene glycol (99%) were bought from Alfa Aesar (Kandel, Germany), and the electrolyte solution was prepared by sodium sulfate (≥99.0%, Sigma-Aldrich, St. Louis, MO, USA). Dimethylformamide (DMF, anhydrous, 99.8%), methanol (≥99.9%), and washing solvent (ethanol and acetone) were all purchased from VWR (Radnor, PA, USA).

### 2.2. Production of TiO_2_ Nanotube Array Electrodes on Ti Foils

The anodization preparation of a 1D structure of TNTA, which has been studied for decades, allows the formation of nanotubes with a diameter of approximately 100 nm and a length that can be varied from 100 nm to 1 mm by controlling the anodization parameters. Before the anodized reaction, titanium foils were ultrasonically cleaned in acetone and ethanol for 20 min sequentially. Then, the clean Ti plates (2.0 × 2.0 cm^2^) were anodized at room temperature in a conventional two-electrode cell with a Pt plate as the counter electrode in ethylene glycol, which contained 2 wt % NH_4_F and 1 wt % H_2_O_2_. A 55 V potential was first applied at an individual duration time of 0.5 h for the corrosion of the Ti plate to form the nanotubes. Then, the anodized Ti plate was ultra-sonicated with ethanol to remove Ti-based residues from the TNTA. After the electrochemical anodization, the samples were rinsed in ethanol and dried in air. The as-prepared amorphous TNTA were annealed in air at temperature of 450 °C for 2 h with a heating rate and cooling rate of 5 °C min^−1^ to convert to the crystalline phase of TNTA into the anatase-type.

### 2.3. Electrodeposition of Ni on TNTA and Bimetallic Fe/Ni on TNTA

First, the as-prepared TNTA (TiO_2_/Ti) electrode was immersed into 40 mL of Ni(NO_3_)_2_ or Ni(NO_3_)_2_/Fe(NO_3_)_3_ mixture electrolyte solution under various molar ratios and operated in the negative potential range of −0.2 to −1.0 V at 10 mV s^−1^ in 2, 5, and 10 cycles by the cyclic voltammetry method. The samples of Ni/TNTA were prepared by the ratios from 0.05 to 0.005 M, and the bimetallic FeNi/TNTA nanocomposites were fabricated at the ratio of 3:2. Then, the Ni or Ni/Fe-deposited TNTA was obtained after drying at 60 °C for overnight, and the hydroxide species of Ni and Ni/Fe would be formed and then deposited onto the surface of TNTA. The electrodeposition was carried out in a conventional electrochemical cell with a three-electrode system, including an Ag/AgCl electrode as the reference electrode (RE) and a graphite electrode as the counter electrode (CE). After electrodeposition, all the electrode was rinsed with absolute ethanol for three times and dried under room temperature.

### 2.4. Transformation of Bimetallic FeNi-MOF/TNTA from FeNi/TNTA

The MOF structure was prepared by transforming the FeNi hydroxide in the presence of organic ligands. First, 0.03 M BTC was dissolved into 30 mL of mixed solution of methanol (MeOH) and DMF (1:9) with continuous stirring for 1 h, and then FeNi/TNTA was face-up put in the bottom of a Teflon tube. Finally, the FeNi-MOF/TNTA was synthesized by hydrothermal reaction under the condition of 150 °C for 4 h. After the hydrothermal reaction, the as-prepared samples were washed by distill water (DI) water and absolute ethanol in sequence for three times before drying at 60 °C for 1 h. The as-synthesized FeNi-MOF/TNTA was cooled to room temperature before the PEC measurement.

### 2.5. Characterization

A scanning electron microscope (FEG-SEM, ZEISS Sigma HD, Munich, Germany) was used for checking the surface morphologies of the different samples. Elemental analysis was performed by energy-dispersive X-ray spectroscopy (EDS) analysis using equipment (SAMx IDfix, Levens, France) fixed on the SEM and operated with an acceleration voltage of 15 kV. An X-ray diffractometer (X’pert pro, PANalytical, Netherlands) with CuKα radiation (wavelength λ = 1.54045 Å) operating at an accelerating voltage of 40 kV and a current of 35 mA in the scan range of 5–80° was used for the identification of the crystallite phases and the structure of TNTA, ZIF-67 MOF, and FeNi-MOF/TNTA-related materials. The chemical states of the different elements were determined by X-ray photoelectron spectroscopy (XPS) analysis using a K Alpha, Thermo Fisher Scientific (Waltham, MA, USA) system equipped with a monochromatic aluminum source (Al Kα, 1486.68 eV). All measurements have been made in an ultra-high vacuum (UHV) chamber (Waltham, MA, USA), at a residual pressure below 10^−9^ mbar. A spot size of 400 µm corresponding to an irradiated zone of approximately 1 mm^2^ was used for the measurements. The hemispherical analyzer was operated at 0° take off angle in the Constant Analyzer Energy (CAE) mode, with a pass energy of 200 eV (and an energy step of 1 eV) for the acquisition of wide scans, against a pass energy of 50 eV (and an energy step of 0.1 eV) for the acquisition of spectra over narrower energy ranges. The charge compensation was achieved by means of a “dual beam” flood gun, using low-energy electrons (5 eV) and argon ions. Samples were fixed on the support using adhesive and conducting tape. The recorded spectra were processed with the Advantage software using a peak-fitting routine with Shirley background and symmetrical 70–30% mixed Gaussian–Lorentzian peak shapes. The atomic ratios were evaluated by normalization of the peak areas with the Scofield sensitivity factors.

### 2.6. Photoelectrochemical Measurements

The optical properties of the different samples were measured using a UV–visible spectrophotometer (Cary 60, Santa Clara, CA, USA). The photoelectrochemical properties of the FeNi-MOF and TNTA-based samples were investigated using a Solartron ModuLap XM potentiostat (Farnborough, UK) and a three-electrode cell equipped with a quartz photoelectrochemical window. Measurements were performed using a working electrode (the prepared samples), a counter electrode (high surface area Pt wire), and a reference electrode (a saturated calomel electrode or SCE). A 0.1 M Na_2_SO_4_ solution at pH 7 was used as liquid electrolyte. Ar gas was bubbled in the electrolyte for 30 min before starting the measurements in order to remove dissolved air. Then, the working electrode was irradiated using a solar simulator (Asahi spectra, MAX-303, Torrance, CA, USA) equipped with a 300 W Xenon lamp. The light intensity of the simulated light was adjusted in order to apply 1 sun (100 mW/cm^2^) over the surface of the working electrode. Linear sweep voltammetry (LSV) was used to record voltammograms at 10 mV/s over the potential range from −0.6 to +1.2 V/SCE under dark and chopped illumination conditions. Chronopotentiometric measurements were made at a constant potential of +1 V/SCE under chopped and continued illumination conditions. Photoelectrochemical impedance spectroscopy (PEIS) was used to record PEIS spectra under UV–visible light, visible light, and dark conditions at different potentials ranging from −0.6 to +1.2 V/SCE, over the 100 kHz to 50 mHz frequency range using an AC voltage amplitude of 10 mV. Mott–Schottky measurements have been made at a constant frequency of 125 Hz over the +1.0 V/SCE to −0.8 V/SCE potential range.

## 3. Results and Discussion

### 3.1. Structural and Morphological Characterization of FeNi-MOF/TNTA

**Figure 1:**
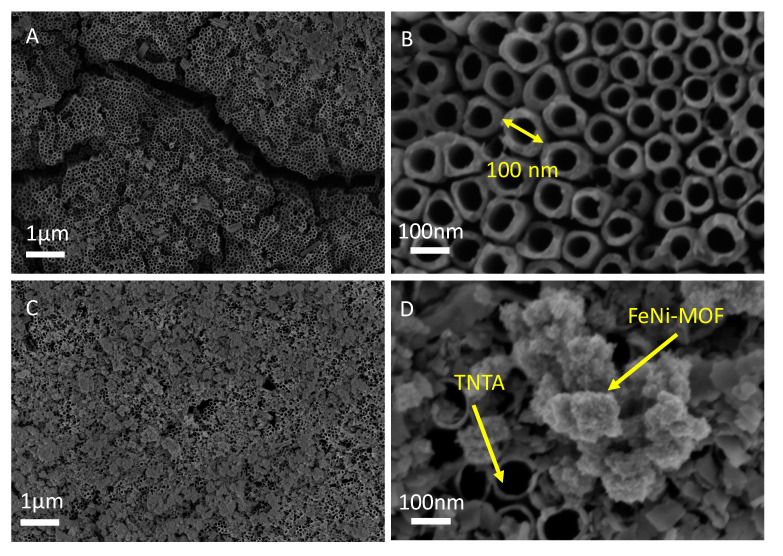
SEM image of (**A**,**B**) anodized TiO_2_ nanotube arrays (TNTA) and (**C**,**D**) nickel and iron bimetallic organic framework (FeNi-MOF/TNTA) (0.03M_Fe/0.01M_Ni) with different magnifications.

The TNTA nanostructure was first observed by SEM at different magnifications which are shown in Figure 1A,B. It is clear that the surface of the Ti foil is not flat, but the tubes structures of TNTA are homogeneous and well-aligned as grown onto the surface of the Ti foil. The diameters and lengths of TNTA are highly dependent on the concentration of the etching agent, operation time, and anodization power of the synthesis method. In this study, the outer diameter of TNTA is about 80–100 nm and the length is around 4.72 µm. In addition, the wall thickness of TNTA is in the range of 8–23 nm, corroborating that the anodization process can produce homogeneous distribution and well-aligned TNTA onto Ti foil. Additionally, in order to reduce defects from the charges flow and the resistance of the interface, the cleaning process is significant before the deposition of MOFs on the surface. The surface was cleaned by ethanol with continuous ultra-sonication for 10 min to make the surface completely spotless without the Ti-based residues. After annealing of TNTA/Ti foil, the as-prepared samples are immersed into 10% HCl solution with ultra-sonication for 10 min to clean the oxidized form of TiO_2_ residues. According to the SEM images of FeNi-MOF/TNTA in Figure 1C,D, the FeNi-MOF nanoparticles were successfully deposited onto the surface of TNTA. The particles of FeNi-MOF are almost around 100 nm, which is similar to the diameter of TNTNA. This means that FeNi-MOF can deposit and cover the top of TNTA. It is noteworthy that the Fe/Ni ratio would influence the coverage of FeNi-MOF onto the TNTA surface. The amount of MOFs under low-loading condition cannot well cover on the top of TNTA, and the Fe/Ni ratio of 3:1 is observed to be optimized to fabricate bimetallic FeNi-MOF for good distribution onto the surface of TNTA. These well-distributed MOFs can support TNTA to enhance the photoactivity (Figure 1C,D).

**Figure 2:**
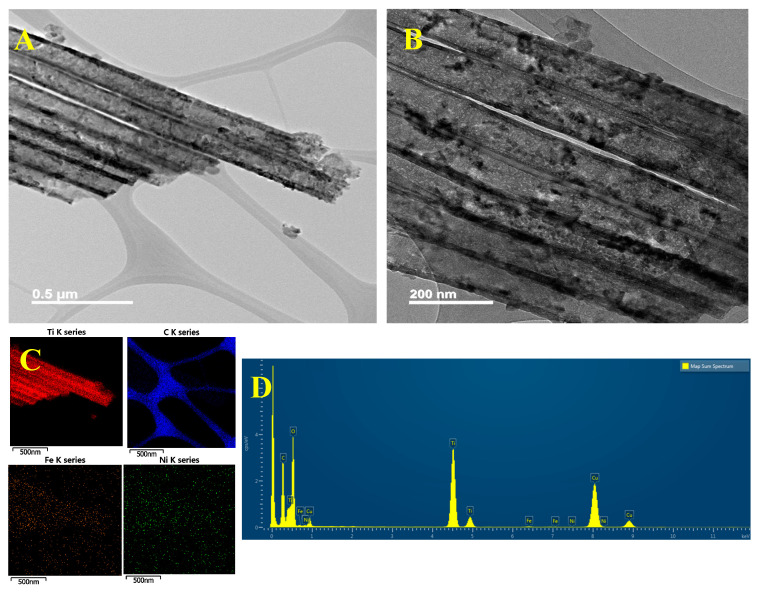
(**A**,**B**) FETEM images with different magnifications, (**C**,**D**) energy dispersive X-ray spectroscopy (EDS) mapping and corresponding spectra of FeNi-MOF/TNTA (0.03M_Fe/0.01M_Ni) nanocomposites.

In order to understand the growth position of MOFs on the TNTA, i.e., in the tubes, out of tubes, or on the top of tubes, FETEM images and EDS mapping were used to examine the morphology and distribution of FeNi-MOF-decorated TiO_2_ nanotubes. The TEM images illustrated in Figure 2A,B with different magnifications show the individual tubes under a cross-sectional view. Some small particles are attached on the surface of TNTA, where the diameter of particles is less than 100 nm. Precisely, the componential elements with a corresponding intensity of FeNi-MOF/TNTA are shown in Figure 2C,D. The elemental mapping of Ti, C, Fe, and Ni indicates that the FeNi-MOF/TNTA contains a high amount of Ti (red points) and less loading of C (blue points), Fe (orange points), and Ni (green points). The total amount of Ti is about 12.9% in the EDS spectrum, but the weight percentages of Fe and Ni are estimated to be less than 0.10%, which is lower than the detection limits of EDS. This means that most of the FeNi-MOFs are grown on the top of TNTA and only little amounts of MOFs are surrounded by TNTA.

**Figure 3:**
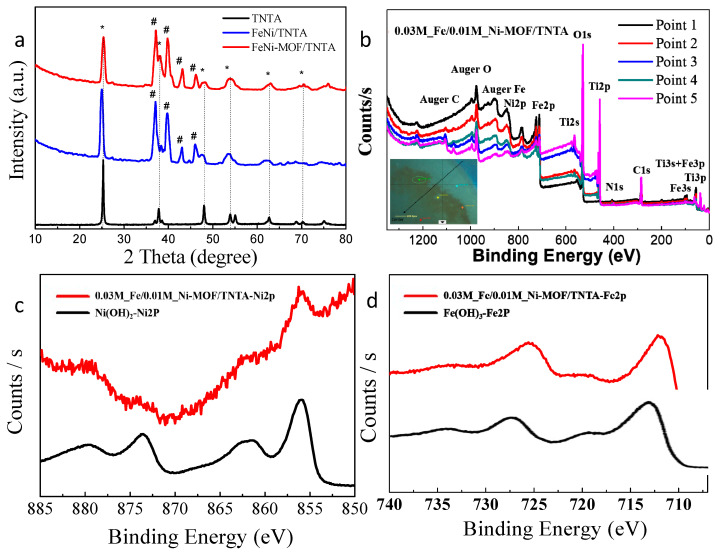
Elemental analysis of the TNTA, FeNi/TNTA, and FeNi-MOF/TNTA (0.03M_Fe/0.01M_Ni) (**a**) XRD patterng (*: anatase; #: Fe/Ni), (**b**) X-ray photoelectron spectroscopy (XPS) survey spectra, XPS spectra of (**c**) Ni 2p, and (**d**) Fe 2p of FeNi-MOF/TNTA.

Figure 3 presents the XRD patterns of TNTA, FeNi/TNTA (0.03 M_Fe/0.01 M_Ni), and FeNi-MOF/TNTA (0.03 M_Fe/0.01 M_Ni) samples. From the XRD patterns, the FeNi-MOF/TNTA shows low crystallinity in comparison with the bare TNTA. The XRD patterns of TNTA exhibit several characteristic crystalline anatase peaks which are located at 2θ = 25.5°, 38.1°, and 48.3° that are identified to be (101), (004), and (200) crystal faces, respectively (JCPDS 21–1272). This means that all the anodized TNTA samples after the annealing at 450 °C over 2 h mainly contain an anatase phase without any evidence on the rutile structure. Since anatase TNTA possesses higher photoactivity compared to rutile TiO_2_ in a PEC system [49], this result suggests the high PEC activity toward OER. In addition, the main peaks with the mark of “#” are FeNi hydroxide at 2θ  = 37.1°, 39.9°, 43.1°, and 46.1°, which are identical to Fe–Ni LDH (JCPDS 74–0748). After the electrodeposition of metals and MOFs growth, the XRD peaks become wide, which may be attributed to the formation of FeNi-hydroxide and FeNi-MOFs. It is worth noting that XRD patterns of FeNi-MOF at 8.8° 2θ are not observed because of the small loading of Fe and Ni contents [46].

In order to understand the elemental species of components in FeNi-MOF/TNTA, the XPS analysis has been measured to confirm the successful growth of FeNi-MOF on the TNTA. Figure 3b shows the survey spectra of the XPS spectra measured on the different locations of 0.03M_Fe/0.01M_Ni-MOF/TNTA sample. The different elemental peaks are indexed as C, O, N, Fe, Ni, and Ti. However, the spectra are not identical in each position of the sample, indicating that the Fe/Ni-MOF is not homogeneously deposited on the TNTA. The dissimilar full XPS spectra are randomly chosen by the laser point on the surface of the sample, and there are some peaks missing such as Ni 2p, Fe 2p, and C 1s (Auger C), and N 1s in points 1 and 4. Undoubtedly, those missing peaks indicate the lack of FeNi-MOF. Additionally, the experimental and referred XPS spectra of Ni and Fe shown in Figure 3c,d, respectively, clearly indicates the existence of Ni and Fe hydroxide. Owing to the concentration and ratio of nickel and iron (Fe:Ni = 3:1), the peak intensity of nickel (Ni 2p) is relatively lower than that of iron (Fe 2p). The peaks at 855.7 and 873.5 eV with satellite peaks at 861.8 eV are the typical Ni^2+^ 2p peak (Figure 3c), while the peaks shown in Figure 3d are the Fe^3+^ peaks located at 711.8 and 725.5 eV, which can be assigned as the Fe 2p_3/2_ and Fe 2p_1/2_ peaks, respectively.

**Figure 4:**
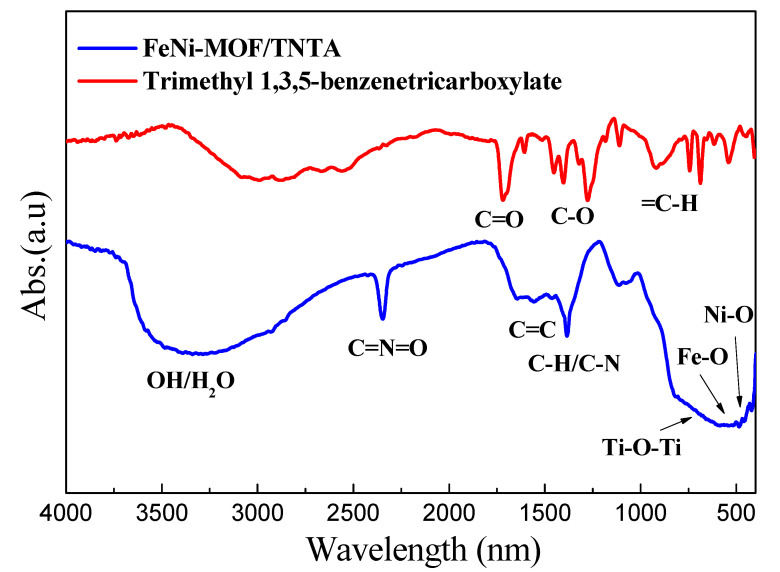
Fourier transform infrared (FTIR) spectra of FeNi-MOF/TNTA (0.03M_Fe/0.01M_Ni), TNTA, and trimethyl 1,3,5-benzenetricarboxylate.

Fourier transform infrared (FTIR) spectra (Figure 4a) shows that FeNi-MOF/TNTA exhibits the typical characteristic peaks for 1,3,5-BTC, which contains –OH, C=O, and aromatic ring function groups. The apparent peak of FeNi-MOF/TNTA firstly observed at 3445 cm^−1^ can be assigned to the bending vibration of –OH or H_2_O on the photocatalyst, and the absorption peaks at 1630, 1584, and 2340 cm^−1^ correspond to the stretching vibration peaks of C=O/C=N, C=C, and C=N=O double bonds. The other peaks located at 3000–3100, 3010–3100, and 1050–1150 cm^−1^ can be ascribed as the aromatic ring of C–H, C=C–H, and C–O stretching vibration. All these peaks are comparable to the same position of the pure BTC organic compound. Additionally, the broad peak between 750 and 400 cm^−1^ is situated as the Ti–O–Ti bonding of TNTA [50], and the other two tiny peaks at 484 and 540 cm^−1^ are related to the metal oxide bond, which belongs to the Ni–O and Fe–O vibration, respectively [51,52]. It is clear that the FeNi-MOF is a kind of cluster-type porous structure, where bimetallic Fe and Ni have successfully linked to the BTC precursor of MOF, which is also proven by XPS results.

### 3.2. Photoelectrochemical Analysis of FeNi-MOF/TNTA

**Figure 5:**
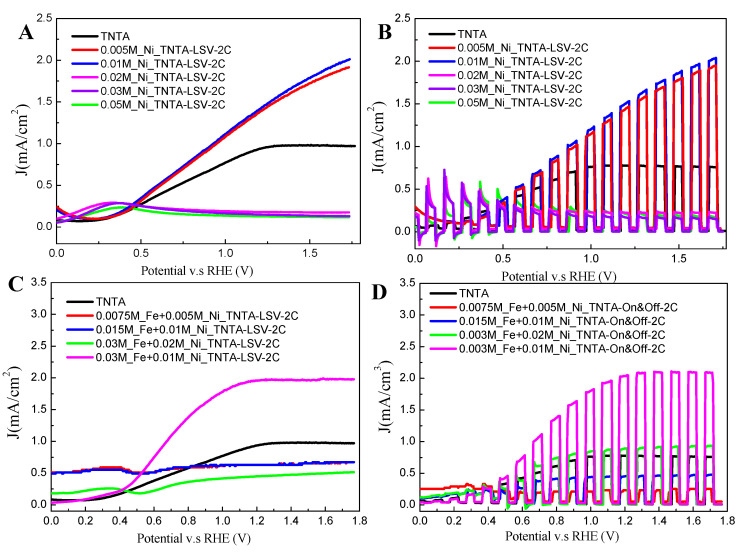
(**A**) Linear sweep voltammetry (LSV) curves of optimization for Ni-TNTA at a scan rate of 10 mV; (**B**) The chopped light chronoamperometric measurements of optimization for Ni-TNTA at a scan rate of 10 mV; (**C**) LSV curves of optimization for FeNi-MOF/TNTA at a scan rate of 10 mV; (**D**) The chopped light chronoamperometric measurements of optimization for Fe/Ni-TNTA at a scan rate of 10 mV.

Photoelectrochemical characteristics of the TNTA and various concentrations of FeNi-MOF/TNTA photoelectrodes are investigated by liner sweep voltammogram (LSV) with chopped UV-Vis irradiation, chronoamperometric measurements, and photoelectrochemical impedance spectroscopy (PEIS) in the PEC water-splitting system. Well-ordered TNTA are prepared and fabricated by the electrochemical anodization on a pure Ti plate (99.9%) in organic solution. All the parameters for TNTA synthesis are related to the photocatalytic performance and conductivity [30]. In this present work, we have fabricated the as-prepared TNTA with the diameter of 100 nm and a length around 4.72 µm under the optimized conditions; therefore, the photo response of 1.0 mA/cm^2^ is obtained during the photocurrent tests. To further understand the effect of FeNi-MOF on the photoelectrochemical performance, FeNi-MOF with various Ni amounts ranging from 0.005 to 0.05 M were electrochemically deposited onto the top of TNTA. As shown in Figure 5A, the LSV curves show that the best current density increase from 1.0 mA/cm^2^ for pure TNTA to 2.10 mA/cm^2^ at 0.01 M Ni under 2 cycles of electrodeposition, and then, the current density decreases dramatically when the Ni amount increases. It is clear that high MOF content induces the contradictory effects, i.e., faster charge transfer kinetics is desirable, while the unfavorable kinetics of charge recombination occurs. Hence, the optimal MOF contents are beneficial to maximize the photocurrent density [22,53]. Similar to the LSV curve operated at a scan rate of 10 mV, the LSV curve with chopped UV-Vis irradiation operated under turn on-off mode in the presence of UV-visible is shown in Figure 5B. When LSV with chopped UV-visible irradiation was used to generate the photocurrent, the photocurrent increases sharply when the UV-visible light is on, and then the current drops to almost zero when light switches off. In addition, all the results are highly dependent on the concentration of Ni(OH)_2_ deposited on TNTA, in which high Ni(OH)_2_ concentration exhibits low photoactivity over photocurrent production. Additionally, the higher loading of Ni (0.02, 0.03, and 0.05 M) enhances another reaction between 0 V and 0.5 V (vs. reversible hydrogen electrode, RHE), which is the phenomena that happens on the redox potential of Ni(OH)_2_. According to previous studies [54], the grand potentials of Ni(OH)_2_ were calculated between 0 and 0.5 V when the grand potential of Ni was set to 0 eV. In order to increase the water-dissociated efficiency at low potential, the bimetallic ions system is first selected and electrodeposited onto TNTA by using Fe(NO_3_)_3_ and Ni(NO_3_)_2_. The results of LSV measurement at different ratios of iron and nickel are shown in Figure 5C,D. It is clear that 0.03 M_Fe/0.01M_Ni/TNTA exhibits the highest performance with a photocurrent density of 2.2 mA/cm^2^ at 1.23 V (vs. RHE). The Fe(OH)_3_ has an important role in enhancing the photocatalytic activity of FeNi/TNTA as well as promoting the water-oxidation process at low potential.

**Figure 6:**
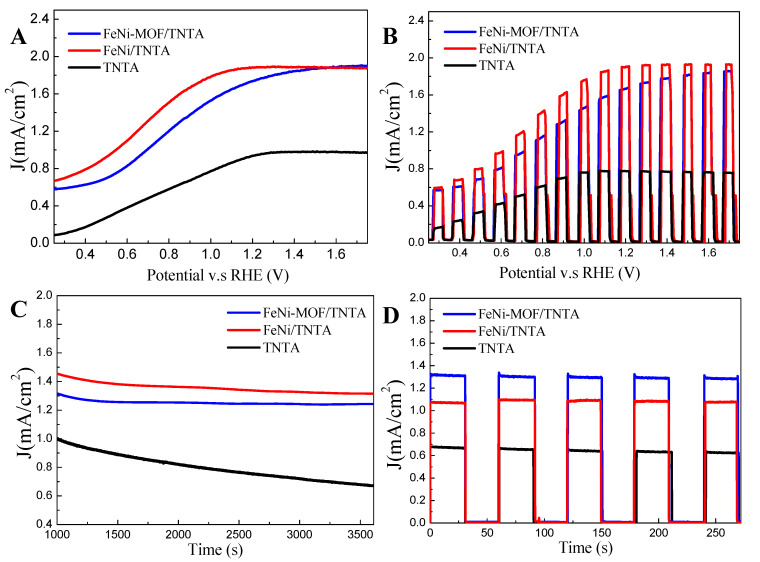
(**A**) LSV curves of optimization for TNTA, FeNi/TNTA, and FeNi-MOF/TNTA at 10 mV; (**B**) The chopped light chronoamperometric measurements of optimization for TNTA, FeNi/TNTA, and FeNi-MOF/TNTA at 10 mV; (**C**) The stability measurement of TNTA, FeNi/TNTA, and FeNi-MOF/TNTA at 1.0 V for 1 h; (**D**) The chopped light chronoamperometric measurements of optimization for TNTA, FeNi/TNTA, and FeNi-MOF/TNTA at 1.0 V after 2 h.

For the purpose of preparation of a highly efficient photoanode for PEC water splitting, the stability of the photosensitizer is the main challenge and breakthrough for long-term operation. Transformation of the MOF structure is a kind of idea to increase the durability of the photoanode when operated at a high potential. Recently, carbon-based architectured MOFs have been considered as a good candidate that can be utilized for heterojunction interface reactions or act as a supporting photosensitizer to promote water dissociation during long-term operation. In this study, FeNi-MOF/TNTA at a Fe:Ni ratio of 3:1 has been successful fabricated and then applied in a PEC system. However, we found that the photocurrent of 1.9 mA/cm^2^ decreases slightly with time, and the dynamic photoresponse is lower than the previous result of FeNi/TNTA as well (Figure 6A,B). For a different perspective, the stability of FeNi/TNTA and FeNi-MOF/TNTA in the first hour is quite stable (Figure 6C), but after 2 h of measurement, the photocurrent of FeNi-MOF/TNTA is higher than that of FeNi/TNTA, as shown in Figure 6D. Moreover, the photocurrent of FeNi/TNTA declines to around 1.1 mA/cm^2^ after 180 min of operation, while FeNi-MOF/TNTA still keeps 1.3 mA/cm^2^ during the measurement. These results clearly indicate that MOF construction contributes high patience and reactivity in PEC water-splitting application. It should be noted that the FeNi oxidation may contribute the photocurrent to the FeNi-MOF. A previous study has reported that the Ni^2+^/Ni^3+^ redox pair can contribute to the Ni/Fe oxidation toward OER at a high voltage of 1.43 V (vs. RHE) [55]. When this situation occurs, the photocurrent would be produced in the dark. In this study, the applied voltage is in the range of 1.0–1.2 V, which is lower than the voltage needed for Ni/Fe oxidation, and the photocurrent can be back to zero when the light is switched off, clearly signifying that the Ni/Fe oxidation has little contribution to the OER activity.

## 4. Conclusions

In this study, the nanoscale FeNi-MOF anchored on a TNTA photoelectrocatalyst has been successfully fabricated through a two-step procedure of electrodeposition and hydrothermal methods. The modified TiO_2_ nanotube array via anodization produces an anatase phase with a diameter of 100 nm and length of 4.72 µm. The pure TNTA exhibits a higher photocurrent behavior (approximately 1 mA/cm^2^) in comparison with the previously reported PEC studies in neutral electrolyte (0.1 M Na_2_SO_4_). After anchoring the bimetallic FeNi-MOF, the FeNi-MOF/TNTA shows the enhanced photocatalytic activity toward OER, and a photocurrent density of 1.9 mA/cm^2^ is clearly obtained when fresh electrode and electrolyte are used. The FeNi-MOF/TNTA still shows high photocatalytic stability (1.3 mA/cm^2^) than FeNi/TNTA (1.1 mA/cm^2^) and TNTA (0.65 mA/cm^2^) after 180 min of measurement, which declines about 31%, 50%, and 35% under the UV-visible light irradiation, respectively. It is clear that the decrease in photoactivity is mainly attributed to the fact that the combination of TNTA with FeNi-MOF significantly improves the charge barriers and electron recombination rate, resulting in the maintenance of its highly photocatalytic property. Henceforth, the asprepared photoanode can be easily reused with excellent stability. These results indicate that the fabricated FeNi-MOF/TNTA is a highly efficient photocatalyst, and it can serve as the promising PEC water-splitting photoanode for OER application under UV-visible light radiation conditions.
